# Detection and Discrimination of Auditory Alerts in Single- and Dual-Task Conditions: Use of a Free-Response Method

**DOI:** 10.1093/milmed/usaf606

**Published:** 2025-12-25

**Authors:** Mabel L Cummins, Morgan Lindstead, Skylar Wechsler, Leslie R Bernstein, Michael Schutz, Joshua Shive, Joseph J Schlesinger

**Affiliations:** Vanderbilt University School of Medicine, Nashville, TN 37212, United States; Department of Anesthesiology, Vanderbilt University Medical Center, Nashville, TN 37212, United States; Northwestern University Feinberg School of Medicine, Chicago, IL 60187, United States; Departments of Neuroscience and Surgery (Otolaryngology), University of Connecticut Health Center, Farmington, CT 06030, United States; School of the Arts, McMaster University, Hamilton, ON L8S 4M2, United States; Department of Psychological Sciences and Counseling, Tennessee State University, Nashville, TN 37209, United States; Division of Critical Care Medicine, Department of Anesthesiology, Vanderbilt University Medical Center, Nashville, TN 37212, United States

## Abstract

**Introduction:**

In military operations, the ability to detect, identify, and respond to auditory alerts in complex and dynamic environments is crucial for safety and mission success. Typical alert designs, however, often fail to account for characteristics of noisy and cognitively demanding conditions, so that the levels of alerts required to support desired levels of performance are minimized. To redress those shortcomings, we developed a pair of alerts, one having consonant harmony (“friendly”), the other dissonant harmony (“enemy”). Those alerts were placed strategically within the spectrum of the masker to minimize masking while maintaining high levels of detection and discrimination performance.

**Materials and Methods:**

The detectability and discriminability of the “friendly” and “enemy” alerts was assessed as a function of signal-to-noise-masker ratio (S/N) while employing a masker consisting of continuous military “truck noise.” Both of the alerts occupied a narrow spectral region within the masker around 500-Hz. Subjects (*n* = 20) performed an auditory detection/discrimination task in isolation or with a simultaneous visual “N-Back task.” The N-Back task was also run in isolation. The auditory task employed a free-response vigilance paradigm with underlying temporal “trials” that were unknown to the subjects. They experienced temporal uncertainty regarding when an alert might be added to the masker. This approach afforded measures of “hit” and “false-alarm” rates and the computation of bias-free measures of sensitivity (*d′*). Trials were blocked by S/N with values of S/N visited via descending and ascending series. Stimuli were presented at an overall level of 70 dB SPL (in the absence of alerts) via Sennheiser HD 280 headphones.

**Results:**

Values of *d′ (sensitivity)* indicated that high levels of detection performance were obtained despite the harmonic “friendly” and inharmonic “enemy” alerts occupying a common spectral locus. That outcome likely occurred because subjects discriminated the alerts on the basis of perceived consonance or dissonance. Values of *ß* (response bias) revealed that subjects adopted conservative response criteria. Turning to discrimination performance, differences between obtained values of *p(c)* and *p(c)_max_* also indicated that subjects did not adopt neutral criteria. In the presence of a simultaneous, visual N-Back task (dual-task condition), auditory detection and discrimination performance was not degraded. In contrast, N-Back performance was poorer in the dual-task condition than when it was measured in isolation.

**Conclusions:**

The results establish “proof of concept” regarding our approach to evaluating detection and discrimination of auditory alerts within a situationally realistic vigilance paradigm. The findings reveal the advantages of employing a Theory of Signal Detection (TSD)-based free-response paradigm to evaluate human performance in such a setting. In addition, the results highlight the potential advantages of employing alerts tailored to the specific spectral profile of the ambient acoustic environment. Overall, our findings can be applied to enhance both the performance and evaluation of practitioners who must respond appropriately to critical alerts in high-consequence settings. The potential enhancements extend beyond military applications, for example, to situations in which clinicians must monitor multiple metrics of patient status in environments with potentially distracting auditory and visual information.

## INTRODUCTION

In military and medical settings, the ability to detect, identify, and respond to auditory alerts in complex and dynamic environments is crucial for safety and success. The design of typical alerts often fails to account for noisy and cognitively demanding conditions that practitioners (e.g., warfighters, physicians) face, whereas taking those conditions into account affords the ability to minimize levels of alerts necessary for desired levels of performance.

Our research focuses on optimizing auditory alerts for cognitively demanding environments, such as theaters of warfare or surgical operating rooms, where frequent sounding of alarms (often more intense than necessary) poses risks to well-being and communication. Of primary importance are the findings of Schlesinger et al.,^1^ who demonstrated that reducing conventional alarm levels below those of ambient noise did not compromise clinicians’ accuracy and speed in identifying alarms, while improving their performance on a secondary auditory task. Complementing those findings, McNeer et al.^2^ explored whether alternative alarm designs could enhance clinical usability even further. Those investigators focused on “auditory icon alarms,” sounds that represent, intuitively, the underlying meaning of a corresponding alarm (e.g., lub dub sound for a cardiovascular aberrancy). They found that auditory icon alarms were “identified more accurately and quickly than the current standard alarms.”^2^ Subsequently, Bruder et al.^3^ explored whether advantages of auditory icon alarms would transfer to multitasking environments. They did. Their findings revealed that, under multitasking conditions, auditory icon alarms yielded greater response reliability and reduced likelihood of missed critical communications than did conventional alarms. To capitalize on these findings, we turned our attention to using novel auditory alerts designed to maximize their detectability and discriminability when presented in a background of military-relevant noise. Those alerts were developed, tested, evaluated, and refined within a preliminary series of pilot experiments.

### Pilot Experiments- Preliminary Testing of Auditory Alerts

We developed 2 pairs of auditory alerts, 1 broadband and 1 narrowband.^4^^,^^5^

### Approach

**Figure 1.**
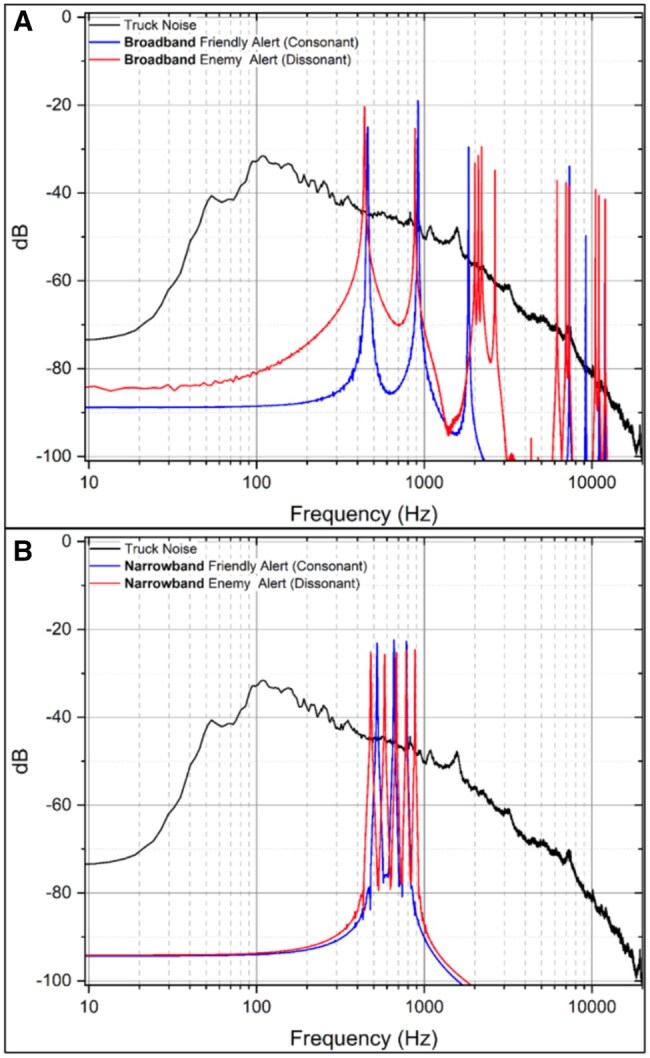
(A) Power spectra of the broadband “friendly” alert (blue), “enemy” alert (red), and “truck noise” masker (black). (B) Same as Panel A, except for the narrowband alerts.

The alerts were masked by continuous “truck noise,” derived from a recording of a military truck in motion. **Figure 1A** and **B** shows the power spectra of the broadband and narrowband alerts and the spectrum of the masker. The blue and red lines indicate the “friendly” and “enemy” alerts, respectively. The black line indicates the power spectrum of the “truck noise.” One member of each pair of alerts was designated as “friendly”; the other member was designated as “enemy.” “Friendly” alerts comprised harmonic components that were perceived as consonant; “enemy” alerts comprised inharmonic components that were perceived as dissonant. The broadband alerts reflected common design practice in that their energy was spread across a wide range of frequencies (see **Figure 1A**). Both narrowband alerts were placed at a common spectral locus (near 500 Hz) within the spectral profile of the masker. That spectral locus occupied a region of relatively low power within the spectral profile of the masker (**Figure 1B**), a choice expected to minimize auditory masking. The expectation was that the narrowband alerts could be presented at low signal-to-noise ratios (S/N) while still yielding high levels of performance. The narrowband alerts were 350 ms long; the broadband alerts were 715 ms long. In the absence of alerts, stimuli were presented via Sennheiser HD 280 Pro headphones at 70 dB SPL.

To measure the detectability and discriminability of the alerts, we conducted a series of pilot experiments, each employing between 14 and 17 subjects, some of whom participated in more than one experiment. In each experiment, we measured psychometric functions for a series of fixed S/Ns. In one series of experiments, subjects were instructed to respond when they *detected* any alert; in another series subjects identified the alert, that is, they *discriminated* between the 2 (“friendly” or “enemy”) when one was detected.

### Findings

The narrowband and broadband alerts yielded robust detection and discrimination, despite the respective pairs of alerts sharing a common spectral locus.^4^^,^^5^ The results obtained with the narrowband alerts, in particular, demonstrate that they can be placed strategically within the spectrum of a masker so as to minimize auditory masking while maintaining high levels of detection and discrimination performance. For a given level of performance, S/Ns only slightly above those required for detection were sufficient for discrimination of the alerts. This suggests that our subjects learned the “meaning” of the alerts by determining whether they were perceived as consonant or dissonant.

### Main Study Objectives

Having the pilot data in hand, we conducted a formal study employing a pair of “friendly” (harmonic) and “enemy” (inharmonic) narrowband alerts as described above. The goal was to assess detectability and discriminability of the alerts under single- and dual-task conditions in complex, multitasking environments. We used narrowband alerts because, in our pilot studies, there was substantial overlap between their psychometric functions relating performance to S/N. They were approximately equally detectable.

## METHODS

### Stimuli

An important feature of the 2 narrowband alerts employed is that they comprised similar spectral components and were both centered in the region of 500 Hz. These choices were made to reduce, if not eliminate, subjects’ use of pitch or temporal cues to identify the alerts.

### Study Population

Via campuswide recruitment, we enrolled 20 Vanderbilt University students, ranging in age from 18 to 27. Thirteen were female; 7 were male. All reported no evidence or history of hearing loss, and normal or corrected-to-normal vision. Subjects gave written informed consent before participation and received compensation after completion of the study. The studies were approved by the IRBs at Vanderbilt University and Vanderbilt University Medical Center.

### Vigilance Task

We employed an objective, free-response vigilance paradigm based on the work of Watson and Nichols.^4^ A fundamental characteristic of the procedure is its use of underlying temporal “trials” that are unknown to the subjects. For the subject, there was temporal uncertainty regarding when an alert might be added to the masker, as is the case with real-world operational environments where alerts can occur at any time. The procedure afforded measures of “hit” and “false-alarm” rates and the objective and bias-free index of sensitivity, *d′*. Those Theory of Signal Detection (TSD)-derived metrics are typically employed with “defined-trial” paradigms. A hallmark of TSD is that it enables investigators to distinguish between a subject’s underlying sensitivity, independent of decision bias.

**Figure 2.**
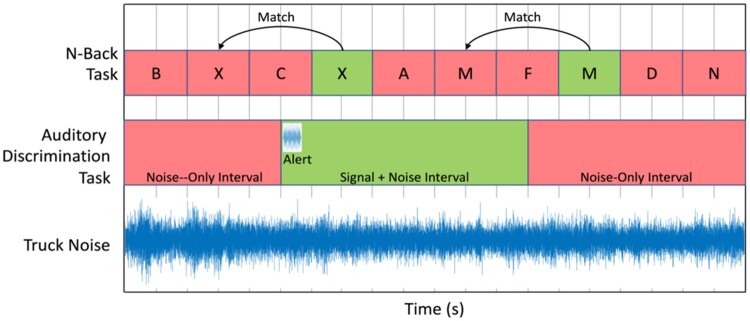
Diagram of the temporal sequence of the auditory discrimination task and the N-Back task.

Our implementation of the free-response paradigm employed random-duration “intervals,” the temporal boundaries of which were inaccessible to the subject. Interval durations were selected randomly from a uniform distribution with a minimum of 5 second and a maximum of 10 second, in steps of 1 second. For each interval, the probability of the occurrence of an alert was 0.5. That is, each of those intervals was assigned randomly as being a signal (alert) + noise or noise-alone interval. During signal + noise intervals, the onset of a 350-ms long “friendly” or “enemy” alert was coincident with the beginning of the interval. “Friendly” or “enemy” alerts occurred with equal probability. For each interval, whether signal + noise or noise-alone, the “response window” began at the onset of the interval and extended 2.35 second. In the case of a signal + noise interval, then, the response window ended 2 seconds after the offset of an alert (the signal). Thus, the response window was identical whether a signal + noise or noise-alone interval had occurred (see **Figure 2**). Only the first response made during a response window was recorded. If that response was recorded during a signal + noise interval, then, independent of whether it was a correct identification (“friendly” or “enemy”), it was characterized as a “hit.” Similarly, if that response was recorded during a noise-alone interval, it was characterized as a “false alarm.” Hit and false alarm rates were used to compute *d′* for detection of the alerts. The metric used to assess discrimination performance (correctly identifying whether an alert was “friendly” or “enemy”) was the probability of a correct identification computed over those intervals during which a “hit” was recorded.

### N-Back Task

We incorporated a visual N-Back task in half of the experimental sessions to simulate an attentionally-demanding environment.^5^ The N-Back task comprises a sequential stream of individual letters appearing at the center of a visual display. Each letter was presented for 200 ms, with no delay between the offset of one letter and the onset of the next. Subjects were instructed to press the “SPACE” bar on a keyboard if the letter appearing was the same as that which had been presented 2 letters earlier in the sequence (see **Figure 2**). Immediately following a keypress, subjects received on-screen feedback indicating whether their response was correct or incorrect.

### Experimental Sessions

Each participant completed 2 separate experimental sessions on consecutive days. The sessions differed only with respect to whether formal data were collected in the auditory discrimination task alone or the auditory discrimination task combined with the N-Back task (dual-task condition). The ordering of the 2 types of sessions was chosen randomly for each subject. Events within each session occurred in the following order: (1) instructions were provided for the N-Back task; (2) the N-Back task in isolation was practiced for 30 seconds; (3) instructions were provided for the auditory discrimination task; (4) the “friendly” and “enemy” alerts were, respectively, identified and played once for the subject; (5) depending on which of the 2 types of sessions was assigned, the subject performed a 30-second-long practice run of either the single- or dual-task condition; (6) formal data were collected during a 5-minute run of the N-Back task in isolation; (7) once again, the “friendly” and “enemy” alerts were identified and played once for the subject; and (8) depending on which of the 2 types of sessions was assigned, formal data were collected for either the single- or dual-task condition.

For the free-response paradigm portion of each session, 8 blocks were run, each at a fixed S/N. Over the first 4 blocks, S/Ns were assigned in descending order. During the next 4 blocks, those same S/Ns were assigned in ascending order. This ordering of S/Ns was implemented to counterbalance any learning or order effects. The fixed S/Ns differed slightly for the “friendly” and “enemy” alerts because our pilot experiments had revealed that the “friendly” alerts were slightly more detectable than the “enemy” alerts for a given value of S/N. By offsetting their respective S/Ns by 1 dB, we achieved near-equal detectability for the 2 types of alerts. For the descending series within a session, the S/Ns for “friendly/enemy” alerts were −10/−9, −12/−11, −14/−13, and −16/−15 dB.

For each of the 4 constant-S/N blocks, there were 10 signal + noise trials and 10 noise-alone trials. Recall that, within each session, 2 blocks of each S/N were run. Thus, each session contained a total of 20 signal + noise and 20 noise-alone trials, with signal + noise trials being divided equally between “friendly” and “enemy” alerts. The overall level of the “truck noise” masker was 70 dB SPL, before the addition of either alert. Stimuli were presented via Sennheiser HD 280 headphones. The stimulus-delivery system was calibrated before each experimental session. PsychoPy version 2023.2.3 served as the software platform.

## RESULTS

### Detection Performance

**Figure 3.**
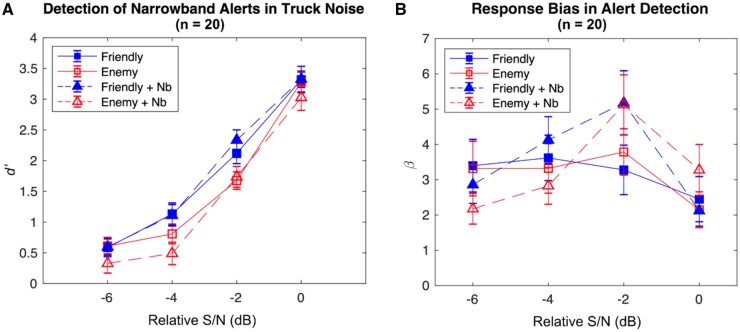
(A) *d′* as a function of relative S/N (dB). (B) *ß* as a function of relative S/N (dB). In both panels, closed and open symbols represent data obtained with “friendly” and “enemy” alerts, respectively. Solid and dashed lines represent data obtained in single- and dual-task conditions, respectively.

For each subject, we calculated *d′* for each combination of S/N and alert identity, in single- and dual-task conditions. On the relatively rare occasions that values of *d′* were calculated to be infinite, all raw counts within the stimulus-response matrix were “corrected” by adding 0.25. Then, *d′* was recalculated (see e.g., Brown and White).^6^ For each experimental condition, we calculated the mean, across subjects, of the individual values of *d′*. **Figure 3A** displays mean *d′* vs. relative S/N. The value of 0 dB corresponds to the highest S/N tested for each type of alert. For the “friendly” and “enemy” alerts, recall that those values of S/N were −10 dB and −9 dB, respectively.

Panel A reveals that, as expected, *d′* increased monotonically with S/N. Overall, “enemy” alerts (open symbols) required levels about 1 dB greater than the “friendly” alerts (closed symbols) for the 2 types of alerts to be equally detectable. Thus, our efforts to achieve essentially equal detectability between the 2 types of alerts were successful. Finally, it appears that the addition of the N-Back task (triangles) to the auditory discrimination task run in isolation (squares) had little, if any, systematic effect on performance. A 3-way repeated measures analysis of variance (ANOVA) using *d′* as the dependent variable with S/N, type of task (single or dual), and alert identity as factors confirmed the effects gleaned from visual inspection. Specifically, there was a significant main effect of S/N (*F*(1,19) = 241.59, *P *< .001, η^2^ = 0.650). The main effect of alert identity was also significant, with higher sensitivity to “friendly” alerts than “enemy” alerts (*F*(1,19) = 41.06, *P *< .001, η^2^ = 0.015). There was no significant main effect of type of task, bolstering the conclusion that there was no “cost” to auditory detection performance as a result of adding the N-Back task.

### Analysis of Response Bias

As conceived within TSD, when participating in detection tasks of the type used here, the observer adopts a criterion for the perceived sensory magnitude above which they respond, “yes,” indicating detection of a signal, and below which they respond, “no,” indicating that no signal was detected. Importantly, the criterion, indexed by the metric, *ß*, is independent of the observers’ “true sensitivity,” indexed by *d′*. A *ß* of 1.0 indicates a “neutral” criterion, reflecting no response bias. Values of *ß > *1.0 indicate conservative criteria (a reduced likelihood of responding “yes”); values of *ß *< 1.0 indicate liberal criteria (an increased likelihood of responding “yes”).

For each subject, we calculated *ß* at each combination of S/N and alert identity, in both single- and dual-task conditions. For each experimental condition, we calculated the mean, across subjects, of the individual values of *ß*. **Figure 3B** displays mean *ß* vs. relative S/N. Note that all values of *ß* plotted are greater than 1.0, indicating that, on average, the subjects adopted conservative criteria in all experimental conditions.

A 3-way repeated measures ANOVA using *ß* as the dependent variable with S/N, type of task (single or dual), and alert identity as factors did not reveal any significant main effects. There was, however, a significant interaction between task and S/N (*F*(3,57) = 2.78, *P *< .05, η^2^ = 0.024). Given the variability observed in **Figure 3B**, and the fact that the interaction accounts for only 2.4% of the total variance in the data, the interaction, while significant, is not, in our view, meaningful.

Next, for each of the 4 experimental conditions, we computed the mean value of *ß* across the 4 relative S/Ns. Then, for each of the 4 resulting mean values of *ß*, we performed a 1-tailed z-score test to evaluate whether, across experimental conditions, the values of *ß* obtained were significantly different than 1.0. We found that all 4 means were significantly greater than 1.0 (*P *= .001) indicating, consistent with visual inspection of **Figure 3B**, that, overall, subjects adopted conservative response criteria.

### Discrimination Performance

We assessed subjects’ abilities to discriminate (identify correctly) the 2 types of alerts by computing *p(c)*, that is, proportion of correct identifications, for each combination of task and S/N. More specifically, we calculated the proportion of trials during which the presence of an alert was both detected *and* identified correctly. Because *p(c)* is a biased measure, we also calculated *p(c)*_max_, the maximum proportion correct that, theoretically, could be achieved assuming that subjects adopted neutral criteria for responding “friendly” or “enemy,” once a target had been detected. To calculate *p(c)*_max_, we again considered only signal (alert) + noise trials and cast the discrimination task as a *signal-detection* task. On trials in which the “signal” was the “friendly” alert, responding “friendly” was coded as a hit, while responding “enemy” was coded as a false alarm. On trials in which the “signal” was the “enemy” alert, responding “enemy” was coded as a hit, while responding “friendly” was coded as a false alarm. This scheme allowed us to calculate *d′* for the discrimination task and, by assuming a neutral criterion, *p(c)*_max_.

**Figure 4.**
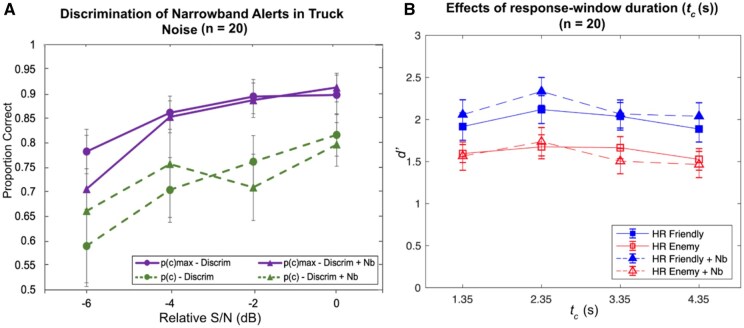
(A) Proportion correct as a function of relative S/N (dB). Dotted lines represent values of *p(c)*; solid lines represent values of *p(c)*_max_. Circles and triangles represent single- and dual-task conditions, respectively. (B) *d′* as a function of *t_c_* (s). Closed and open symbols represent data obtained with “friendly” and “enemy” alerts, respectively. Solid and dashed lines represent data obtained in single- and dual-task conditions, respectively.

If subjects’ obtained values of *p(c)* fell below *p(c)*_max_, that would indicate that neutral criteria had not been adopted. The data plotted in **Figure 4** are mean values of *p(c)* and *p(c)*_max_ computed across subjects. The dotted lines in Panel A represent the values of *p(c)*; the solid lines in Panel A represent the values of *p(c)*_max_. Panel A shows that, as expected, *p(c)* and *p(c)*_max_ increased monotonically with S/N. Note that the values of *p(c)* are, for all conditions, about 10% to 15% below their corresponding values of *p(c)*_max_. This indicates, once again, that subjects, overall, did not adopt neutral criteria. Thus, the biased dependent variable, *p(c)*, underestimated underlying sensitivity in the discrimination task, while *p(c)*_max_, by definition, did not.

A 2-way repeated measures ANOVA with S/N and dependent variable (*p(c)* or *p(c)*_max_) confirmed the trends observed in **Figure 4A**. The main effect of S/N (*F*(3,57) = 5.71, *P *< .01, η^2^ = 0.045) was significant as was the main effect of dependent variable (*F*(1,19) = 9.99, *P *< .01, η^2^ = 0.044).

### Effect of Response Window Duration on d′

Recall that the duration of the “response window” in our free-response paradigm was 2.35 second. We refer to that duration, as did Watson and Nichols, as *t_c_*. Watson and Nichols^4^ suggested that the optimum value of *t_c_* would be that resulting in the maximization of estimates of detection sensitivity. Accordingly, we re-computed estimates of *d′* for the auditory detection task using values of *t_c_* equal to 1.35, 2.35, 3.35, or 4.35 seconds, at a relative S/N of −2 dB for both single- and dual-task conditions. The results are presented in **Figure 4B**. The data reveal 2 trends: (1) values of *t_c_* ranging between 1.35 and 4.35 seconds yielded only modest differences in *d′*; (2) as seen in **Figure 3**, values of *d′* were elevated for “friendly” versus “enemy” alerts.

A 2-way repeated measures ANOVA with *t_c_* and alert type as factors was conducted. There was a significant main effect of *t_c_* (*F*(3,57) = 3.38, *P *= .02, η^2^ = 0.012). Note that the main effect accounted for only about 1% of the variance in the data. There was also a significant main effect of alert identity (*F*(1,19) = 19.52, *P *< .001, η^2^ = 0.099), reflecting, once again, that the “friendly” alerts were slightly more detectable than were the “enemy” alerts. Pairwise comparisons revealed that values of *d′* were significantly higher when *t_c_* = 2.35 second compared to when *t_c_* = 4.35 second. Likewise, *d′* values were significantly higher when *t_c_* = 3.35 second than for *t_c_* = 4.35 second. No other main effects or interactions were significant. Again, despite these significant statistical outcomes, **Figure 4B** makes apparent that, for each alert type, estimates of *d′* based on values of *t_c_* between 1.35 and 4.35 second are, for practical purposes, essentially identical. Thus, it appears that choice of *t_c_* = 2.35 second in our full analyses did not underestimate subjects’ overall sensitivity.

### N-Back Task Performance

We compared N-Back performance between single- and dual-task conditions. In this case, the single task was the 5-minute-run of the N-Back task in isolation (see “Experimental Sessions”). For each participant, we calculated an estimate of *d′* for the single-task condition by computing the mean of the *d′* obtained for each of the 2 experimental runs of that condition. The results indicate that performing the N-Back task concurrently with the auditory discrimination task (dual-task condition) degraded N-Back performance as compared to when that task was run in isolation. A paired-samples *t*-test of the mean of the individual estimates revealed that *d′* was higher when the N-Back task was performed in isolation than when that task was performed simultaneously with the auditory discrimination task (*t*(19) = −8.71, *P *< .001, *d* = −1.95). Recall that, in contrast, the inclusion of the N-Back task did not degrade auditory discrimination performance. Taken together, these results suggest that the subjects prioritized the auditory task by focusing attention on it at the expense of the N-Back task.

## DISCUSSION

Our results represent “proof of concept” regarding our approach, experimental techniques, and stimulus design. The findings demonstrate the validity and utility of our free-response vigilance paradigm emulating conditions encountered by practitioners who must respond appropriately to critical auditory alerts in high-consequence settings. Such settings involve temporal uncertainty regarding *when* a “signal” might occur. Our vigilance task, *from the point of view of the listener*, preserved that uncertainty while incorporating an underlying trial structure such that TSD-based, bias-free measures of observer sensitivity and bias could be obtained. That our subjects, overall, adopted conservative response criteria, means that, had we employed biased measures, then their abilities to detect and discriminate the alerts would have been underestimated.

High levels of detection and discrimination performance were maintained by placing the alerts strategically within the spectrum of the masker to minimize auditory masking. Those high levels of discrimination performance were obtained despite the “friendly” and “enemy” alerts occupying a common spectral locus. Apparently, our subjects could discriminate the alerts based on their perceived consonance or dissonance.

In dual-task conditions, a simultaneous visual N-Back task did not degrade auditory performance. On the other hand, the presence of the auditory discrimination task did degrade N-Back performance as compared to that obtained with the N-Back task in isolation. It appears that subjects prioritized the auditory task at the expense of the N-Back task.

Having validated our approach in terms of the psychophysical procedure and the design of auditory alerts, the next step would be to employ broad samples of military personnel as subjects within “high-fidelity” simulated acoustic environments of incrementally increasing realism. Those broad samples would, by design, yield heterogeneity in terms of hearing status (e.g., those with normal hearing, slight losses, moderate losses, etc.). It would be important to evaluate performance in our free-response vigilance paradigm as a function of hearing status.

Furthermore, future experiments should explore the relative efficacy of the intuitive types of auditory alerts we have designed vs. more conventional military and medical alerts. Our laboratory has, in fact, made such comparisons.^1^^,^^3^ Those studies demonstrated that, compared to conventional alerts, “auditory icon” alerts could be presented at substantially lower overall levels without degrading performance and afforded improved performance on both auditory and visually-based tasks when subjects were required to attend to both types of tasks simultaneously.

## CONCLUSIONS

This study establishes “proof of concept” regarding our methodology designed to evaluate auditory alert detection and discrimination using a “realistic” paradigm mimicking high-consequence settings in which practitioners must respond appropriately to critical auditory alerts that can occur at any time. Bias-free measures of sensitivity, derived from the Theory of Signal Detection, revealed that our subjects adopted conservative response criteria.

In our view, the demonstrated advantages of a free-response paradigm that affords calculation of TSD-based metrics suggests that it would be prudent to employ such a paradigm within future studies evaluating human performance under realistic vigilance conditions. It appears that using auditory alerts tailored to specific acoustic environments such that masking can be minimized, while allowing those alerts to be discriminated along intuitive perceptual dimensions (e.g., consonance versus dissonance), represents a useful tool in the design of alerts to be employed in real-world settings.

Before real-world implementation of our design, it would be necessary to conduct a series of experiments employing broad samples of military personnel, both normal hearing and those with loss as subjects within “high-fidelity” simulated acoustic environments of incrementally increasing realism.

## Data Availability

The data supporting the findings of this study are available upon request from the corresponding author.
